# Mitochondria: the epigenetic regulators of ovarian aging and longevity

**DOI:** 10.3389/fendo.2024.1424826

**Published:** 2024-11-13

**Authors:** Shalini Mani, Vidushi Srivastava, Chesta Shandilya, Aditi Kaushik, Keshav K. Singh

**Affiliations:** ^1^ Centre for Emerging Diseases, Department of Biotechnology, Jaypee Institute of Information Technology, Noida, India; ^2^ Departments of Genetics, Dermatology and Pathology, Heersink School of Medicine, University of Alabama at Birmingham, Birmingham, AL, United States; ^3^ Center for Women’s Reproductive Health, Heersink School of Medicine, University of Alabama at Birmingham, Birmingham, AL, United States

**Keywords:** epigenetics, menopause, ovary, aging, mitochondria

## Abstract

Ovarian aging is a major health concern for women. Ovarian aging is associated with reduced health span and longevity. Mitochondrial dysfunction is one of the hallmarks of ovarian aging. In addition to providing oocytes with optimal energy, the mitochondria provide a co-substrate that drives epigenetic processes. Studies show epigenetic alterations, both nuclear and mitochondrial contribute to ovarian aging. Both, nuclear and mitochondrial genomes cross-talk with each other, resulting in two ways orchestrated anterograde and retrograde response that involves epigenetic changes in nuclear and mitochondrial compartments. Epigenetic alterations causing changes in metabolism impact ovarian function. Key mitochondrial co-substrate includes acetyl CoA, NAD+, ATP, and α-KG. Thus, enhancing mitochondrial function in aging ovaries may preserve ovarian function and can lead to ovarian longevity and reproductive and better health outcomes in women. This article describes the role of mitochondria-led epigenetics involved in ovarian aging and discusses strategies to restore epigenetic reprogramming in oocytes by preserving, protecting, or promoting mitochondrial function.

## 1 Introduction

The decline in reproductive aging is one of the main major risk factors associated with women’s health and higher morbidity and treatment-related adverse events for numerous aging-related anomalies (1, 2). Ovarian aging affects women’s reproductive function. Indeed, ovarian aging is linked to 22 age-related illnesses, which can be roughly classified into metabolic, cardiovascular, orthopedic, cancer, and neurological/cognitive disorders (3). Expanding recent research has shown that epigenetic modifications, in addition to genetic variables, are crucial for the onset and advancement of ovarian aging (1).

Epigenetic changes are inherited changes and without affecting the original DNA make-up, epigenetic modifications could lead to changed gene expression. These epigenetic changes are reported in both nuclear as well as mitochondrial DNA (mtDNA) though the understanding of mtDNA epigenetics has only recently gained recognition (4). Both these genomes communicate with each other and thus generate a coordinated bi-directional anterograde and retrograde gene response involving epigenetic processes (5).

Altered mitochondrial function influence ovarian function (6) via epigenetic modifications (7). The nuclear genome contains dynamic epigenetic marks, and a healthy mitochondrial metabolism is required to supply the intermediary metabolites required for the production and alteration of these epigenetic markers and thus regulate gene expression. Key metabolites that serve as co-substrates for epigenetic activities, include ATP, α-ketoglutarate (α-KG; also known as 2-oxoglutarate, 2-OG), β-nicotinamide adenine dinucleotide (NAD+), and acetyl coenzyme A (acetyl CoA). These metabolites are provided by mitochondria (8). Mitochondrial dysfunction (such as due to the reduction in mtDNA content and ATP levels) has been suggested as one of the characteristics of aging, and there is mounting evidence linking it to ovarian aging (6, 9). Further research into how to preserve, protect, and promote mitochondrial function in aging ovaries is needed to develop mitochondrial therapeutics for ovarian longevity to improve women’s reproductive health and reduce the risk of menopause-associated diseases.

This article primarily highlights the role of mitochondria in the epigenetic regulation of ovarian function and aging and provides strategies to prevent or delay the risk of major health issues associated with reproductive aging in females. We propose that a deeper comprehension of the crucial responsibilities, mitochondria play in regulating epigenetics in the ovaries, may lead to clinical advances in preventing or delaying ovarian aging. We describe approaches to target the epigenetic reprogramming in oocytes, to reduce the associated health risks.

## 2 Ovarian function and factors responsible for its aging

Ovarian aging is described as a biological loss of ovarian function, which results in menopause and infertility (10). Ovarian aging is characterized by a progressive decline in the number and quality of the follicles in the uterus. Unlike men, who reproduce indefinitely, women have few primary follicles that begin to decline as soon as they are born (11). Menopause occurs in most women about age 51 years of age; early menopause occurs before the age of 45 (12). Fertility is significantly impacted by the depletion of oocytes available for ovulation and fertilization. Consequently, menopause is a critical stage of reproductive aging that lasts for several decades and impacts women’s health (2).

**Figure 1:**
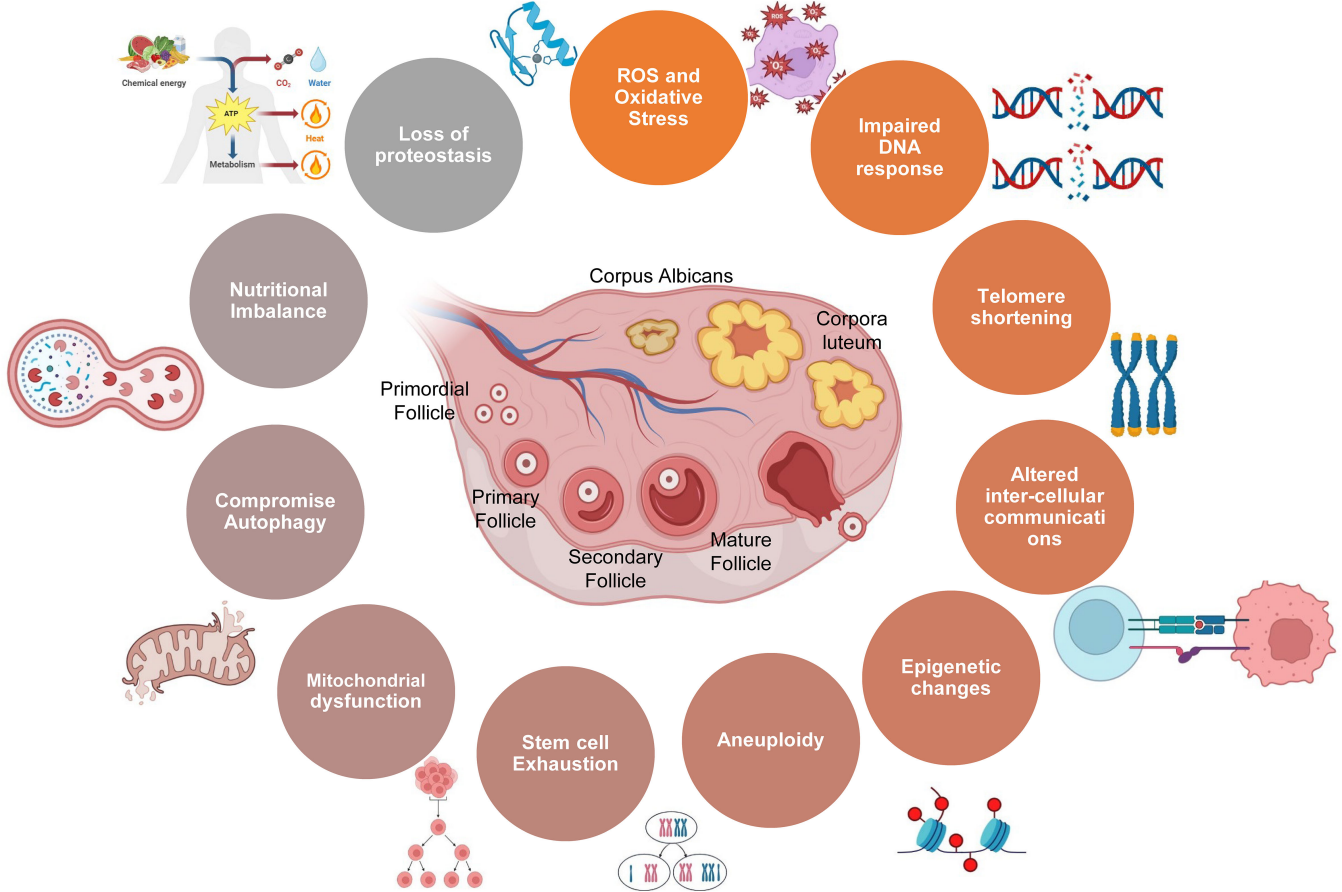
Schematic showing various factors involved in ovarian aging.

As women navigate the diverse stages of life, their reproductive journey unfolds against the backdrop of intricate biological processes, genetic influences, and environmental factors. The gradual decline in ovarian function and fertility is underpinned by mitochondrial dysfunction, genomic instability, oxidative stress, genetic alterations, aneuploidy, epigenetic changes, gradual reduction in telomere length, senescence, stem cell depletion, and disrupted intercellular communication (1, 6). Together, these factors collectively contribute to the diminishing ovarian aging and reproductive health with advancing age, as summarized below (**Figure 1** **).**

### 2.1 Oxidative stress

Even though reactive oxygen species (ROS) are necessary for the physiological function of the ovary such as follicular growth and ovulation, higher levels of ROS are associated with cellular dysfunction and biomolecule damage, a major factor in ovarian aging. Studies indicate the age-related accumulation of ROS-induced biomolecular damage to DNA, lipids, and proteins in ovarian cells (13). Furthermore, the interaction of the biomolecules with ROS generates harmful products which further induce oxidative damage to the ovarian micro-environment. Advanced glycation end-products (AGEs) are one such product, generated through irreversible non-enzymatic interactions between amino groups in proteins, lipids, and nucleic acids with reducing carbohydrates like glucose. Oxidative damage is believed to be caused by the interaction between AGE and the AGE receptor (RAGE) (13). Furthermore, AGEs aggravate oxidative stress in the ovary by interacting with ROS and causing oxidative damage to the ovarian microenvironment. ROS and AGE interactions are linked to ovarian aging and reproductive dysfunction because AGEs activate proinflammatory pathways, degrade oocyte quality, and cause oxidative stress in the ovary. Interactions between AGE and RAGE receptors may also affect oocyte quality and vascular endothelial growth factor (VEGF) production, which may affect the outcomes of *in vitro* fertilization (IVF) and other reproductive treatments. Together, oxidative stress and AGE decrease oocyte function as quality, which contributes to ovarian aging. Thus it is critical to maintain a redox balance for reproductive health (10). Overproduction of ROS reduces antioxidant reserves, leading to oxidative stress, damage to DNA, and early aging (14). Another study found that long-term ROS exposure in mice reduces oocyte quality and fertility (15). Antioxidant therapy such as N-acetyl-L-cysteine (NAC) can reduce ROS damage, improve oocyte quality, and extend reproductive longevity (15).

### 2.2 Impaired DNA repair responses linked to genetic alterations and nutritional imbalance

Ovarian aging is regulated by the intricate relationship between genetic variables and DNA repair mechanisms. Genetic factors influence the age at which natural menopause (NM) begins. Over 100 of the genes like FMR1, MYADML, BRCA1/2, MCM8, POLG, NOBOX, PTEN, FIGLA, FOXO3, GPR3, POF1B, etc. have been associated with the start of premature ovarian insufficiency (POI) (16) (14). Recent studies including genome-wide association studies (17–19) also suggest the role of metabolic signaling networks and DNA damage response (DDR) pathways, in controlling ovarian aging. Studies indicate that early menopause and reduced oocyte reserves are also observed to be associated with dysfunctional DNA repair of mutated BRCA1 and BRCA2 genes, otherwise commonly linked to breast cancer (20–22). Ovarian aging is also accelerated by abnormalities in checkpoint signaling pathways. For instance, cell cycle checkpoint kinase 2 (CHK2) is vital for responding to DNA double-strand breaks (DSBs) and variations in CHK2 have been linked to NM (23). This highlights the significance of preserving genomic integrity to sustain ovarian function and fertility throughout the course of a female’s lifetime (14).

One of the top ranking reproductive disorder is POI and nutrients imbalance can cause such disorder. The components of nutrients found in daily meals include vitamins, carbohydrates, fat and lipoprotein, protein and polypeptide, and fruits and vegetables that contain phytoestrogens. Because of their proliferative, anti-inflammatory, antioxidant, and mitochondria protective properties during the menopausal transition, these nutrients are considered to be important factor (24).

### 2.3 Telomere shortening and aneuploidy

One of the effects of cell division is the shortening of telomeres, which are protective caps on chromosomes. Telomeres that are too short can abuse cell dysfunction and encourage oocyte death (11). The telomere length affects the onset of menopause as well as influences reproductive lifespan, making it an important factor in the advancement of ovarian age. Studies have indicated that women with shorter telomeres are linked to premature menopause and infertility, whereas those with longer telomeres usually have longer fertility and delayed menopause (25–27). Ovarian telomere attrition is caused by many factors such as oxidative stress and decreased telomere activity, which result in chromosomal abnormalities, aneuploidy in embryos, and decreased ovarian reserve (14, 28–30).

Aneuploidy, or an inappropriate number of chromosomes in oocytes, impacts the development of the embryo and results in miscarriage or implantation failure, especially in older women (31, 32). An increased risk of aneuploidy in aged oocytes is caused by discrepancies in chromatin segregation during meiosis I and II, spindle impairments, and improper kinetochore attachments (33, 34). A further risk factor is the age-related decrease in cohesiveness between sister chromatids and homologous chromosomes (34, 35). Improper chromosome segregation is made more difficult by the presence of univalent, which is the result of premature bivalent segregation (34, 36). These results highlight the intricate processes that underlie aneuploidy in mature oocytes (14).

### 2.4 Stem cell exhaustion

The supply of proliferating follicles is replenished by ovarian stem cells. Age-related decreases in the biomass of these stem cells can further reduce oocyte production (37). Our understanding of adult ovarian biology has been completely transformed by the identification of oogonial or germline stem cells (OSCs) in female mammals, which suggests a possible treatment for ovarian aging. These adult stem cells remain tissue-specific and have been the subject of several studies on a variety of mammalian species, including humans. These studies have revealed their characteristics and functions, including the ability to produce viable offspring both *in vitro* and *in vivo* through the fertilization of functioning oocytes. Recent research suggests OSC population heterogeneity may vary as they become more mature, maybe as a result of oxidative damage or genetic abnormalities (38). Despite significant debate regarding the OSC extraction process, studies have confirmed the existence of OSC smaller populations expressing certain surface markers, such as CD61 and ALDH activity (39, 40). Transcriptional profiling also identifies processes that are evolutionarily conserved and underlie the inactivity of OSC with aging, providing insight into possible targets for ovarian aging mitigation (37).

### 2.5 Altered intercellular communication

For the oocyte to function properly, communication between different cell types within the ovaries is important. Age-related alterations in this communication have the potential to impair oocyte health and follicular development (16). Alterations in cell connections during follicular development play a significant role in the etiology of both primary ovarian insufficiency (POI) and polycystic ovary syndrome (PCOS). Patients with PCOS have decreased expression of growth factors like GDF-9 along with altered gap junction (GJ) activity in cumulus-oocyte complexes (COCs), which may be a factor in abnormal follicular development. Moreover, in PCOS and POI, communication may be impaired by the refutation of transzonal projections (TZPs) during ovulation, which is controlled by the receptor for the epidermal growth factor signaling pathway (41). Oocyte competency and quality are also impacted by aging-related alterations in cellular communications in follicles. Findings on long non-coding RNAs (lncRNAs) in follicular fluid have shown that exosomes mediate intercellular communication, which provides insights into the pathophysiology of PCOS. The results of assisted reproduction may be improved by increasing cell interaction in developed follicles through approaches like collagenase therapy (42). Additionally, single-cell sequencing studies open up new research directions by illuminating the molecular dynamics driving follicular development and related disorders (43–45).

### 2.6 Epigenetic changes

Changes that affect the expression of genes without changing the DNA pattern itself are known as epigenetic changes that affect gene expression patterns essential to oocyte growth and function (11). Complex epigenetic modifications that go beyond sequence changes in DNA involve gene silencing, DNA modifications, and the impact of non-coding RNAs (ncRNA). Enzymes such as DNMT1, DNMT3A, and DNMT3B regulate DNA methylation, which is necessary for gene expression and cellular processes and may be involved in the aging process of the ovaries (10, 23, 46). Age-related alterations in histone modifications, such as acetylation and methylation, affect gene regulation and chromosomal integrity. Furthermore, ovarian diseases like PCOS and POF, follicle growth, and oocyte maturation are related to ncRNAs (47, 48). Though research on a variety of mammalian species has shown their varied roles and potential as therapeutic targets, their specific significance in natural ovarian aging is unclear (10). Such changes and their effects will be discussed in later sections.

### 2.7 Mitochondrial dysfunction, autophagy and loss of proteostasis

Mitochondria that produce energy are multifunctional organelles (6). As a hallmark of aging, mitochondrial dysfunction impairs oocyte development (49). Mitochondrial dysfunction can lead to aneuploidy because of abnormal chromosomal segregation during cell division and irregular spindle formation during fertilization. Impaired embryonic mitochondrial inheritance can affect the quality of oocytes and compromise female fertility (49).

Mitochondrial dysfunction also includes impaired autophagy, which is associated with ovarian aging and affect the health of oocytes. It is essential for eliminating damaged mitochondria and preserving oocyte quality for mitochondria to undergo autophagy, particularly via the PINK1-Parkin pathway and receptors such as BNIP3, NIX, and FUNDC1. Reduced fertility is partly caused by age-related abnormalities in this biological process. Improving mitochondrial autophagy can lead to better reproductive outcomes and higher-quality oocytes. Accurately quantifying mitochondrial autophagy is difficult, however. Overall, ovarian aging is associated with a decrease in mitochondrial quality control, including autophagy. This reduction in mitochondrial function and energy supply has an effect on the health of oocytes. Furthermore, in naturally aged mouse oocytes, the loss of proteostasis can impair microtubule stabilization and meiotic integrity, exacerbating the oocyte quality reduction (50)

## 3 Role of mitochondria in ovarian function

**Figure 2:**
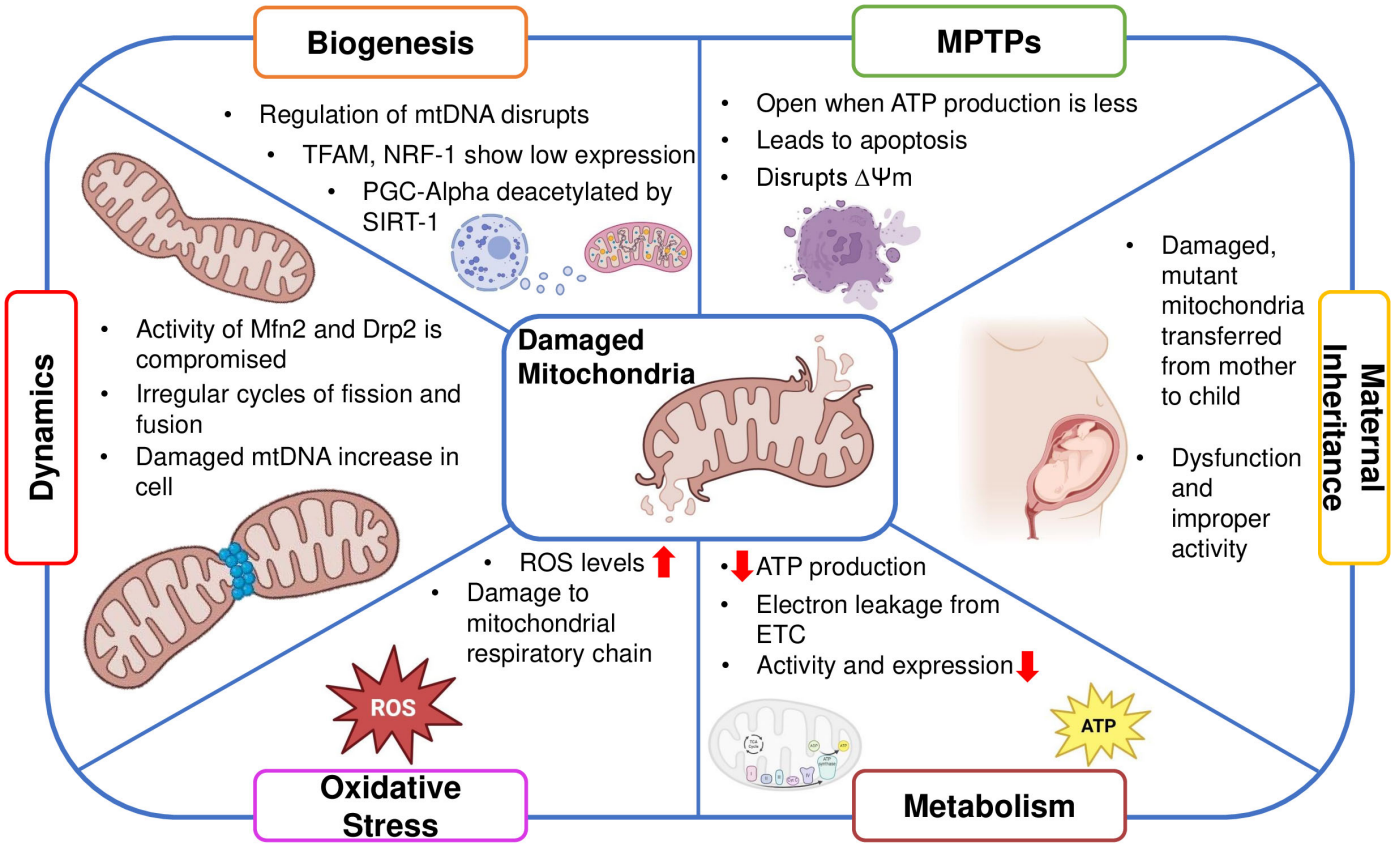
Schematic showing the significance of mitochondrial activity for ovarian function.

Besides many functions mitochondria support the energetic demands of ovarian cells, impacting oocyte maturation and overall fertility. Dysregulation of mitochondrial function influences ovarian health and reproductive outcomes (6, 51) The reproductive senescence, marked by the depletion of ovarian follicles includes the oocytes’ apoptosis as well as the follicular cells around them (52). Mitochondria, known to regulate apoptosis (53), are centrally involved in follicular atresia (54). In addition, the generation of steroid hormones like progesterone and estrogen, which are essential for luteal support, ovulation, and follicle formation, is also tightly regulated by mitochondria. Thus, healthy mitochondrial activity is important for overall ovarian function and longevity (**Figure 2** **).**

Mitochondrial dysfunction in oocytes disrupts ATP supply, impairing meiotic progression and increasing the risk of chromosome segregation errors, leading to aneuploidy. It also alters metabolic pathways, affecting nutrient uptake and biosynthesis critical for oocyte growth (55). Consequently, oocytes with impaired ATP production exhibit reduced developmental competence, resulting in decreased rates of fertilization, poor quality of the embryo, and compromised implantation potential (49) The majority of ROS produced internally, a hazardous end outcome of OXPHOS, are produced by dysfunctional mitochondria. This occurs when electrons are leaking from the respiratory chain pathway, causing accumulation of free radicals and disrupting the healthy functioning of mitochondria (56) Oocyte quality and ovarian function are impacted by damaged mitochondria resulting in a decline of mtDNA content and reductions in ATP synthesis (57, 58). Moreover, deterioration of mitochondrial function and a reduction in mitochondria-produced ATP transferred through mitochondrial permeability transition pores, which is triggered by oxidative stress, can promote spindle disassembly in mouse oocytes, a prominent factor leading to aneuploidy (59).

Additionally, the disruption of the membrane potential of mitochondria and dissemination of apoptotic factors contribute to cell death (60). This compromises cellular function, leading to impaired follicle development and decreased oocyte quality, contributing to declining fertility. Understanding mtPTPs could help develop interventions to preserve ovarian function and extend reproductive lifespan (61). A study demonstrated that ROS production and mPTP opening were increased in the premature ovarian failure group. As a result of this dysfunction in mitochondria, granulosa cell apoptosis occurred which increased the rate of aging in the ovaries (62)

Mitochondria are dynamic organelles that undergo constant fission and fusion. Large dynamin GTPases (DRP1, OPA1, MFN1, and MFN2) anchored in mitochondrial membranes mediate mitochondrial dynamics. Smaller mitochondria are produced by fission, which may promote cell division but also raise ROS levels. Fusion, on the other hand, lessens the aggregation of mtDNA mutations and damaged proteins by fostering communication between mitochondria and other cellular constituents (63). For organelles to closely complement one another and meet cellular energy needs, mitochondrial fusion is necessary (64). Ovarian function can be significantly impacted by mitochondrial proteins, like Dynamin-related protein 1 (Drp1) and Mitofusin 1 (Mfn1). While Drp1 deletion causes oocyte maturation problems that affect oocyte quality (65), Mfn1 deletion speeds up the loss of ovarian follicles (66). Premature ovarian insufficiency (POI), and normal ovarian aging demonstrate decreased mitochondrial biogenesis (6, 41)

Mitochondrial biogenesis ensures a sufficient amount of energy for implantation and the growth of the embryo (65, 67). The transcription factor A of mitochondria is primarily responsible for regulating the expression of mtDNA. TFAM, a ubiquitous transcription factor that targets the mitochondria and promotes mtDNA transcription and replication, and by respiratory factors 1 and 2 of the nucleus (NRF1 and NRF2), which regulate mtDNA expression and the expression of the respiratory chain components encoded by the nucleus. This ensures the essential association between the nuclear and mitochondrial genomes (68).

Dependent on NAD+, silent information regulator 1 (SIRT1) becomes an important regulator of mitochondrial dynamics. Throughout follicle development it regulates mitochondrial biogenesis, protects against oxidative stress, and upholds energy balance (69) Peroxisome proliferator-activated receptor-gamma co-activator 1 alpha (PGC1α) is deacetylated by SIRT1, highlighting its significance in regulating mitochondrial activity. TFAM is a downstream target molecule of the SIRT1/PGC1α pathway. Through the AMP-activated protein kinase (AMPK) pathway (70, 71), SIRT1 further modifies the mitochondrial landscape within ovarian granulosa cells by deacetylating PGC1α. This modulates the quality and function of mitochondria (72, 73).

Cumulus granulosa cells (cGCs) depend on mitochondria for vital energy for both growth and metabolic processes (74). In addition to supplying energy, mitochondria have a strong influence over important biological processes like autophagy, apoptosis, and antioxidant defense, all of which are vital for reproductive processes (75). Because they are the main originator of reactive oxygen species (ROS), they have a significant effect on important reproductive parameters like granulosa cell proliferation, follicular development, and oocyte quality (76). Research shows that females with decreased ovarian reserve (DOR) have lower expression of PGC-1α in oocyte and cumulus cells and lower activity of Sirtuin 3 (SIRT3) compared to those with normal ovarian reserve. This emphasizes a potential connection between subpar oocyte quality in IVF treatments and impaired mitochondrial biogenesis during oocyte maturation (41)

During egg development, mitochondrial biogenesis in the ovaries rises, guaranteeing enough ATP for fertilization and embryonic development. Poor oocyte quality may be a result of reduced mitochondrial biogenesis (77). In mouse models, for example, the lack of Caseinolytic Peptidase P (Clpp), a mitochondrial matrix peptidase that is responsible for quality control and proteostasis of mitochondria, causes an early reduction in ovarian follicles; this may be because the mTOR pathway is activated more than usual (78). This mTOR pathway causes aberrant mitochondrial biogenesis and replicates damaged mitochondria altering their function and in turn altering the reproductive functions. Using processes like Clpp-mediated proteostasis, mitochondria also govern the regulation of protein quality. Ovarian age is influenced by genetic differences in mtDNA. Addressing infertility and age-related ovarian decline requires an understanding of these mitochondrial processes (72).

Maternal transmission of mitochondria is a common process across various organisms, predominantly facilitated by the exclusive transmission of mtDNA through oocytes (79, 80). Over time, as women age, mitochondrial function declines. This deterioration in mitochondrial function impairs oocyte competence and follicular function, contributing significantly to the reduction in ovarian reserve and fertility observed with advancing maternal age (81). Mutations have been discovered through studies in mitochondrial DNA that could be connected to the emergence of PCOS (82)

In addition to determining the age of oocyte development and ovarian aging, mitochondria also affect the quantity and quality of ovocytes (83). Comprehending these pathways is crucial to formulate tactics to prevent untimely ovarian aging; yet, creating pharmacological remedies to revitalize mitochondria in this situation is still difficult. There are several implications associated with the abnormal functioning of mitochondria. Any anomaly can cause defects in the genetic, metabolic, and epigenetic factors associated with ovarian function.

## 4 Epigenetics of ovarian aging

Ovarian aging is significantly influenced by epigenetic modifications, which influence on expression of gene and cellular processes essential for ovarian function and reproductive health (84, 85). Age-related reduction in ovarian function can be attributed to dysregulated expression of genes inculpated in follicular development, steroid formation, DNA repair, and apoptosis caused by changes in epigenetic markers (1, 86) The quality of oocytes generated by the ovaries is influenced by epigenetic variations (87) Aging oocytes with aberrant epigenetic patterns increase the risk of infertility (88, 89), miscarriage, and birth abnormalities by causing genomic instability, mitochondrial dysfunction, and changed gene expression depiction (90, 91). These conditions also compromise the quality of the oocytes (92).

**Figure 3:**
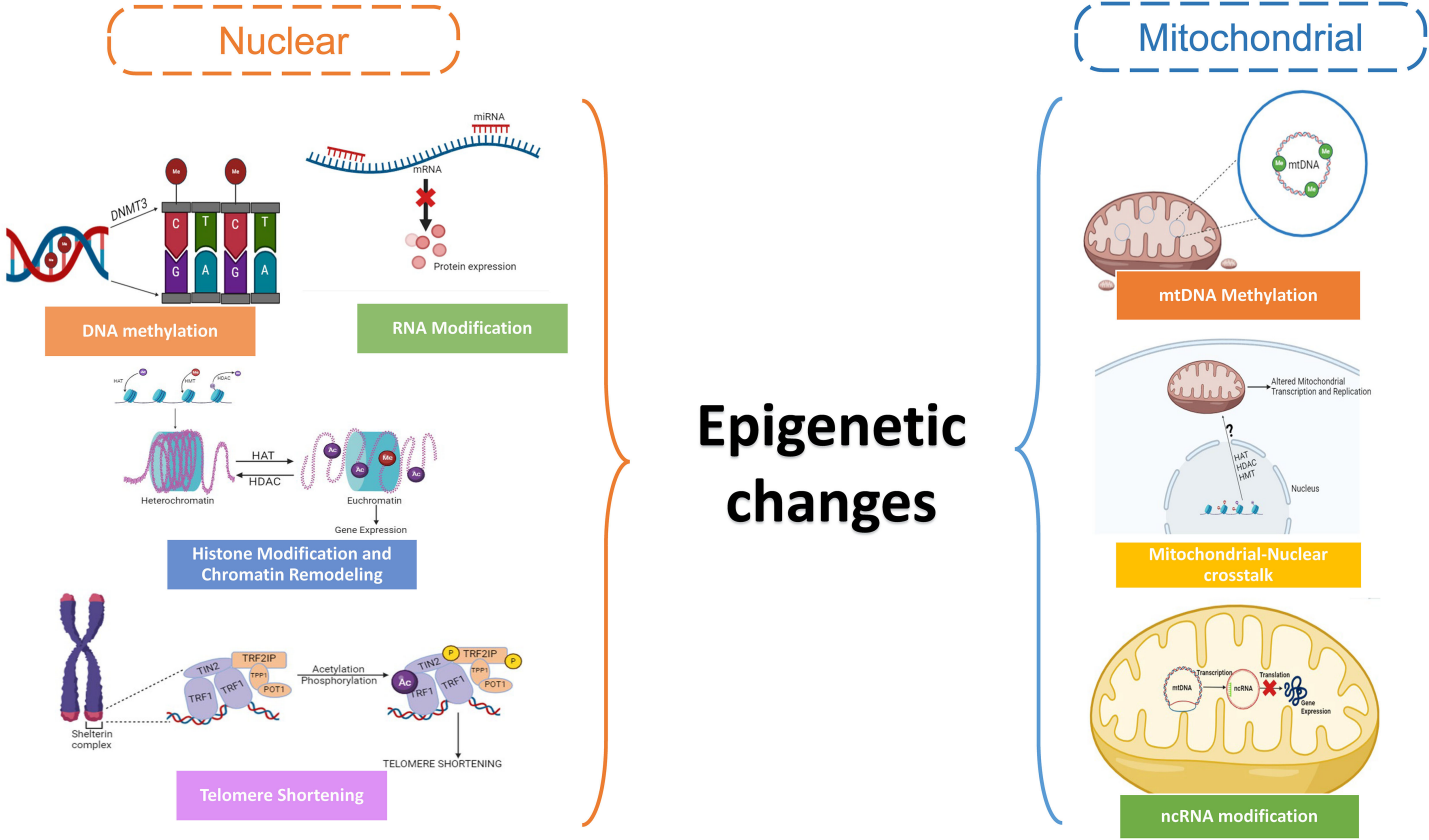
Schematic showing the epigenetic changes in the nuclear and mitochondrial genome in oocytes.

Reduced reproductive capacity and infertility arise from dysregulation of epigenetic pathways by altering the biological age, which can also speed up the onset of menopause, and hinder follicle recruitment and maturation (93, 94). By modifying the expression of genes that promote apoptosis or minimizing the expression of proteins that inhibit apoptosis in granulosa and oocyte cells, epigenetic modifications can accelerate follicular atresia (95). The ovarian reaction to environmental stimuli, such as exposure to endocrine-disrupting substances, stress, and nutritional variables, is also mediated by epigenetic changes (96). Damage from the environment can cause epigenetic changes in the ovaries, which can interfere with normal gene expression patterns and cellular processes linked to ovarian aging and declining fertility (97). The epigenetic changes in the nuclear and mitochondrial genome are responsible for altering ovarian function and reproductive longevity (**Figure 3** **).**

### 4.1 Epigenetic clocks

The simple unit of measurement for time, chronological age, is the number of years that an individual has been alive from birth (98). But throughout our lives, our body undergoes certain biological changes determined by various biological markers and functional abilities of the body that comprise the biological age of an individual (99). We can determine the biological age of an individual by analyzing the pattern of DNA methylation (as discussed later in the section) via the concept of epigenetic clocks. These clocks are predicated on the hypothesis that specific DNA methylation patterns undergo predictable changes with aging, mirroring cellular aging (100). Researchers can create models or algorithms that estimate a person’s biological age by examining these patterns. Numerous epigenetic clocks have been created to estimate the age of the ovaries and forecast the length of the reproductive life (101). By including nuclear epigenetic modifications, these clocks offer insights into the rate of ovarian aging and possible declines in fertility. The Horvath clock, created by Steve Horvath in 2013, is one of the most widely used epigenetic clocks. It is based on DNA methylation levels found by genome-wide DNA methylation profiling at specific CpG sites throughout the gene pool. The Horvath clock is renowned for its accuracy in estimating biological age across a range of populations and is relevant to a range of tissues and cell types. It has also demonstrated the ability to forecast several age-related events, such as the probability of death and susceptibility to disease (102). In 2013, another epigenetic clock was created by Gregory Hannum called the Hannum clock. It uses DNA methylation patterns to measure biological age, just like the Horvath clock. However, because the Hannum clock is specifically trained on blood sample methylation data, it’s especially applicable to research involving blood-based biomarkers. The Hannum clock is associated with the risk of mortality and age-related disorders, and it has been demonstrated to accurately predict chronological age (103). Newer addition to this, second-generation clocks have come into consideration. Developed by Morgan Levine in 2018, the GrimAge clock improves biological age prediction by incorporating other aging-related biomarkers beyond DNA methylation. The GrimAge clock takes into account plasma protein levels linked to aging, including metabolic health indicators and inflammatory markers, in addition to DNA methylation patterns. This multifaceted method improves age prediction accuracy and sheds light on the molecular mechanisms underlying aging and diseases associated with aging (104). Another epigenetic clock called the PhenoAge clock was created to calculate biological age and forecast health outcomes connected to aging. Unlike conventional clocks, it includes DNA methylation data together with clinical indicators including blood cell counts, renal function, and inflammatory markers (105). These epigenetic clocks have been an asset in obtaining key findings on how epigenetic changes affect aging (106).

### 4.2 Epigenetic changes in the nuclear genome

When women age, their ovaries’ nuclei change their epigenetic architecture. These changes are known as nuclear epigenetic modifications in ovarian aging. DNA methylation, histone modifications, chromatin remodeling, non-coding RNA regulation along with telomere dynamics are among the nuclear epigenetic changes associated with ovarian aging (107).

#### 4.2.1 DNA Methylation

The process of adding methyl groups to the residues of cytosine in CpG dinucleotides is known as DNA methylation. Gene expression profiles change when ovarian aging occurs due to changes in DNA methylation patterns (108). Genes essential for ovarian function may be silenced by hypermethylation of promoter regions, whereas genes linked to aging may be abnormally activated by hypomethylation (5). During oocyte maturation, DNA methylation in the female germline of mice is reprogrammed, mainly by enzymes called DNA methyltransferases 3 (DNMT3s) (109). But, during oogenesis- the development of egg cells, both internal and external variables (9) have the potential to stop this process of epigenetic reprogramming. For instance, it has been demonstrated that maternal obesity and diabetes in mice change the DNA methylation patterns in oocytes, which may have an impact on the well-being of the offspring. Likewise, variations in DNA methylation across different organs and disease susceptibility have been connected to anomalies in one-carbon metabolism that are implicated in DNA methylation (110).

#### 4.2.2 Histone modification

Multiple post-translational changes of histone proteins, such as the processes of acetylation, methylation, phosphorylation, and ubiquitination, affect the chromatin structure and the expression of genes. Histone changes in ovarian aging can impact chromatin accessibility to transcriptional machinery, which in turn can control the gene expression profiling related to hormone synthesis, cellular senescence, and follicular development (111). Aging is also linked to changes in histone modification levels, including H3K9me3 and H3K9ac, in both living things and lab conditions. These changes are controlled by enzymes like histone methyltransferases (HMTs), histone demethylases (HDMs), histone acetyltransferases (HATs), and histone deacetylases (HDACs) (112). HDACs are categorized into classes I, II, III (sirtuins or SIRTs) (113) (114), and IV, whereas HATs are grouped into type A and B superfamilies. Important epigenetic enzymes involved in oocyte aging that shield germ cells from oxidative damage include SIRTs, particularly SIRT1. By controlling the chromatin state, lowering oxidative stress, and preventing age-related illnesses, SIRT1 maintains genomic integrity. In aged oocytes, SIRT1, 2, and 3 restore aberrant mitochondrial distribution, mostly by controlling ROS. In addition, SIRT1 activity rises with aging in reproductive cells, delaying DNA damage and senescence (115). Reducing ovarian reserve or prolonged maternal age in women can have an impact on the ovarian microenvironment due to the regulation of metabolic processes by SIRT3 and SIRT5, which are localized in mitochondria (116)

Studies indicate that the regulation of longevity and aging is influenced by H3K4me3, which is linked to active gene expression, and H3K27me3, which is linked to gene silencing. While H3K4me3 levels can change with age based on the species and environment, H3K27me3 levels show distinct patterns in many organisms (117).

#### 4.2.3 Chromatin remodeling

One of the main reasons for ovarian aging is alterations in chromatin structure. Changes in the compactness and arrangement of chromatin as women age might affect DNA accessibility to factors associated with transcription and regulatory proteins, which can affect the expression of genes (118). The aging-related reduction in ovarian function and fertility may be attributed in part to dysregulated chromatin remodeling. The control of gene expression during critical procedures such as corpus luteum development, ovulation, oocyte maturation, and the process of follicle formation and development depends on chromatin remodeling (119). The expression of particular genes at particular periods closely controls these activities, and chromatin remodeling helps to modulate gene expression by facilitating or obstructing other protein regulators and transcription factors access to the DNA (120).

The structure of chromatin in the ovaries is altered by several chromatin remodeling complexes and enzymes, including chromatin remodeling complexes dependent on ATP (like SWI/SNF, ISWI, and CHD complexes) (121, 122) and histone modifiers (like histone acetyltransferases, histone deacetylases, histone methyltransferases, and histone demethylases. Histone methyltransferases (HMTs) (123) and UHRF1 (124) are examples of epigenetic factors that interact with DNA replication machinery components, like the Proliferating cell nuclear antigen (PCNA) clamp (125), to repair chromatin structure after replication. Histone proteins can have chemical changes added or removed by these enzymes and complexes, changing the approachability of DNA to transcriptional machinery. By these pathways, chromatin remodeling regulates gene expression, which is important for ovarian development, hormone signaling, follicle growth, ovulation, and other activities that are necessary for healthy ovarian function. Infertility, PCOS (polycystic ovary syndrome), and ovarian cancer are among the reproductive illnesses that can result from the dysregulation of chromatin remodeling processes in ovaries (126).

#### 4.2.4 RNA Modifications

Without changing the DNA sequence, epigenetic modifications in RNA are chemical changes that take place post-transcriptionally and control gene expression and other biological functions. The most common change is mRNA alteration, N6-methyladenosine (m6A), which affects translation, splicing, and RNA stability.5-methylcytosine (m5C) is a component of several RNA species, it regulates translation, affects RNA stability, and determines the fate of cells. RNA stability, translation efficiency, and structure are all impacted by N1-methyladenosine (m1A). Pseudouridine (Ψ) is abundant in non-coding RNAs and affects the structure and function of RNA. On ribose sugars, 2’-O-methylation (2’-O-Me) takes place, which influences the stability of RNA and its interactions with proteins. Adenosine-to-inosine (A-to-I) editing, a process that is mediated by ADAR enzymes, modifies the sequence and structure of RNA and has an impact on the production and function of proteins. These alterations play key roles in development, cellular homeostasis, and the pathophysiology of illness by dynamically regulating RNA processing, localization, and function (127). Non-coding RNAs control the transcriptional and post-transcriptional stages of gene expression but do not code for proteins. Examples of these RNA molecules are microRNAs (miRNAs) and long non-coding RNAs (lncRNAs) (128). Non-coding RNAs can interact with chromatin-modifying complexes within the nuclei of ovarian cells, thereby regulating gene expression patterns that are necessary for follicular development, oocyte maturation, and overall ovarian function. The age-related loss in ovarian function and fertility can be attributed to the dysregulation of non-coding RNAs in ovarian aging, which can affect gene expression networks (129)

#### 4.2.5 Telomere dynamics

At the tip of chromosomes, repeated DNA sequences called telomeres shield the ends against fusion and breakdown. Because of the end replication issue, telomeres shrink within the nucleus of ovarian cells with every cell division (130, 131). A protein complex called shelterin, attaches to telomeric DNA to shield telomeres against deterioration and improper DNA repair procedures. Telomere function and stability are impacted by epigenetic alterations, such as protein phosphorylation and acetylation within the shelterin complex, which control the complex’s interactions with telomeric DNA and related components (132). Cellular senescence and aging are linked to telomere shortening. Telomere dynamics in ovarian aging can affect follicular cells’ ability to replicate, which can decrease follicle formation and reduce ovarian reserve.

### 4.3 Epigenetic changes in mitochondrial genome

The epigenetic management of mitochondrial DNA (mtDNA) and mitochondrial function within ovarian cells varies as women age, a process known as mitochondrial epigenetic modifications in ovarian aging. Although mitochondria have DNA that is distinct from nuclear DNA, they can experience epigenetic changes that can affect how they function and speed up the aging process in ovaries (4) Recent research has revealed low amounts of methylation in mtDNA by using techniques like bisulfite sequencing (133) and methylated DNA immunoprecipitation (MeDIP) (134), especially in the regulatory D-loop region, despite the genetic material being long thought to be unmethylated. The control of mitochondrial transcription and replication has been linked to methylation of mtDNA. Mutations in mtDNA methylation patterns may arise with ovarian age, impacting the expression and function of mitochondrial genes. Age-related ovarian function reduction and mitochondrial malfunction have been linked to altered mtDNA methylation (135). The expression and function of mitochondrial genes can be impacted by modifications to the packing and structure of mitochondrial DNA. Nucleoids, which are arranged into nucleoprotein complexes from mitochondrial DNA, are crucial for transcription and mtDNA preservation (136). Ovarian cells may have mitochondrial malfunction and reduced energy production as a result of changes in nucleoid structure and organization in aging mitochondria, which may affect the efficiency of replication and transcription of the mtDNA (137).

Despite the absence of histones in mitochondria, there is evidence that histone-modifying enzymes encoded in nuclear DNA localize to mitochondria and may alter the mtDNA’s epigenetic state (138). Mitochondrial transcription and replication can be influenced by histone changes which can impact mitochondrial function. A variety of non-coding RNAs, such as long non-coding RNAs (lncRNAs) and microRNAs (miRNAs), are said to be linked to the regulation of mitochondrial function (139). Aging tissues have been shown to exhibit dysregulation of ncRNAs, and it is conceivable that changes in ncRNAs that target mitochondria may be a factor in the malfunctioning of mitochondria during ovarian aging (140). There are various metabolic components of the mitochondria that contribute to the regulation of the above processes, potentially reducing the effect of these epigenetic changes.

## 5 Mitochondrial metabolites regulate epigenetic changes

**Figure 4:**
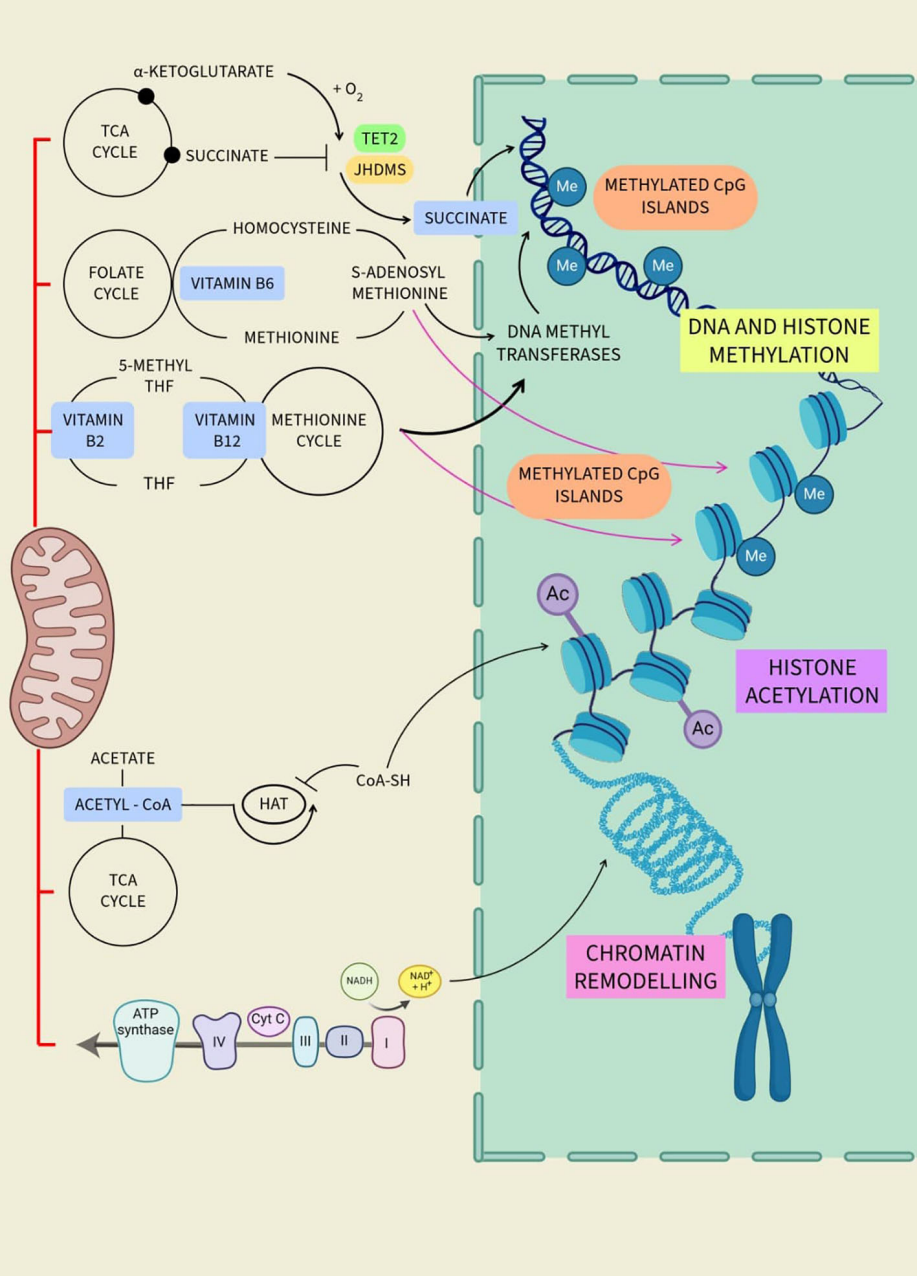
Regulation of different epigenetic processes by mitochondrial metabolites.

Mitochondrial metabolites play an important role in cellular function and influence epigenetic alterations. These metabolites, such as acetyl-CoA, alpha-ketoglutarate, and S-adenosylmethionine, serve as cofactors and catalyst substrates for enzymes that modify DNA and histones, thereby affecting gene expression (**Figure 4** **).** Alterations in mitochondrial metabolite levels can impact follicular development, oocyte maturation, and overall ovarian function. Dysregulation of these metabolites may contribute to conditions such as polycystic PCOS and POI. Therefore, studies aiming to understand the interplay between mitochondrial metabolites and epigenetic modifications can offer insightful information on ovarian function, aging, and longevity (**Table 1** **).**

**Table 1:** Studies summarizing the role of mitochondrial metabolites in regulating the epigenetic changes.

S No.	Epigenetic metabolites	Pathway generating the metabolite	Role in regulating Epigenetic changes	Significant outcomes	References
1	Vitamin B9 (Folate)	Folate cycle (cytosol and mitochondria of the cell)	Due to the folate cycle, a methyl group is donated to homocysteine, converting it into methionine.	Short-term folate depletion can reduce global DNA methylation and upturn might be possible with folic acid supplementation	(147, 148)
2	Vitamin B12	Folate and methylation cycle (cytoplasm)	Coenzyme for methionine synthetase	Supplementation of B12 and folic acid resulted in changes in DNA methylation of several genes, including DIRAS3, ARMC8, and NODAL genes. However, studies on premenopausal women show no correlation.	(168)
3	Vitamin B6	Trans-sulfuration pathway	Methylation of homocysteine	Studies on premenopausal women show no correlation.	(169)
4	Vitamin B2 (Riboflavin)	Folate cycle (cytoplasm)	Precursor for flavin adenine dinucleotides, which act as a cofactor of MTHFR, (catalyzes the reduction of 5,10-methylene THF to 5-methyl THF)	Studies on premenopausal women show no correlation.	(169)
5	Betaine	Transmethylation cycle	Betaine also regulates homocysteine levels, crucial for preventing its accumulation and associated health complications	Lack of studies to understand the effect on ovarian aging.	(170)
6	Choline	Transmethylation cycle	Choline oxidation to betaine facilitates the maintenance of methionine homeostasis, crucial for DNA methylation reactions.	Choline proved a positive influence on ovarian function *in vivo*.	(171)
7	Methionine	Methionine-homocysteine cycle	The availability of methionine influences the production of SAM and subsequently impacts DNA methylation levels	Significantly positive association between metabolic age and methionine, in human follicles. It also included age-associated changes in biomarkers	(172)
8	Acetyl-CoA	Kreb’s cycle	Regulates the activity of TET enzymes	Lack of studies to understand the effect on ovarian aging.	(141).
9	Acetyl-CoA	Kreb’s cycle	Acetyl-CoA acts as a common donor of the acetyl group. In the presence of HATs, the acetyl group is transferred to the ϵ-amino group of lysine side chains. Of histone proteins.	Although studies regarding ovarian aging are not available, acetyl-CoA has been linked to histone acetylation in hypothalamic inflammation, a driver of aging.	(156)
10	Acyl-CoA	Kreb’s cycle	Acyl-CoA molecules act as signaling molecules and directly influence acylation dynamics in chromatin	Acyl-CoA synthetase long-chain family member 6 (ACSL6) gene has been observed to be correlated with POF.	(8, 173)
11	NAD+	Multiple processes	Regulates the activity of sirtuins	Relationship between a decline in NAD+ levels and POF	(159)

### 5.1 Mitochondrial metabolite regulating DNA methylation

Ovarian aging is a multidimensional process marked by declining fertility and alterations in oocyte quality (106). Central to this phenomenon are epigenetic modifications, particularly DNA methylation, which wield significant influence over gene expression during pivotal stages of ovarian development. Metabolites, small molecules deeply entrenched in cellular processes, serve as crucial substrates or cofactors in the intricate machinery governing DNA methylation. Grasping the profound impact of these metabolites on ovarian aging offers valuable insights into potential avenues for therapeutic intervention.

DNA methylation, a fundamental epigenetic mechanism, entails the addition of methyl groups to cytosine residues within CpG dinucleotides (141). This biochemical modification primarily occurs at gene promoters and regulatory regions, exerting profound effects on gene expression dynamics. Within aging ovaries, perturbations in DNA methylation patterns emerge, affecting genes crucial for oocyte development, follicular growth, and hormonal regulation.

At the forefront of DNA methylation regulation stands DNA methyltransferase 1 (DNMT1), an enzyme pivotal for preserving methylation patterns. DNMT1 utilizes S-adenosylmethionine (SAM) as a substrate to transfer methyl groups onto the DNA molecule, a process that generates S-adenosylhomocysteine (SAH), subsequently converted to homocysteine (Hcy) (142, 143). This enzymatic cascade ensures the faithful replication of methylation patterns during DNA replication, safeguarding epigenetic inheritance. Beyond DNMT1, a cadre of other enzymes orchestrates the dynamic regulation of DNA methylation patterns. DNMTs, including DNMT3a and DNMT3b, serve as *de novo* methyltransferases, laying down methyl marks during embryogenesis and cellular differentiation (144, 145). Their concerted actions establish tissue-specific methylation patterns and fine-tune gene regulatory networks critical for ovarian function. In a 2020 study, Uysal and Öztürk analyzed the ovaries of mice at various stages of aging, including prepubertal, pubertal, post-pubertal, and aged (146). Their findings indicated a consistent and significant decline in the expression of DNMT1, DNMT3a, and DMNT3l global DNA methylation patterns, as well as genes at the mRNA and protein levels, as the mice transitioned from youth to old age. These observations led them to infer that the notable changes in the expression of DNMT and levels of global DNA methylation during the process of ovarian aging could potentially be a contributing factor to the onset of infertility in females in their later years.

The ten-eleven translocation (TET) dioxygenases emerge as key players in DNA demethylation, oxidizing methyl groups of cytosine residues. This passive demethylation process, occurring during DNA replication, facilitates the reintroduction of unmethylated cytosines into the DNA molecule, contributing to epigenetic plasticity. Regulation of TET enzyme activity hinges upon AMP-activated protein kinase (AMPK)-mediated phosphorylation, sensitive to cellular energy status and glucose levels. Perturbations in AMPK signaling, particularly under hyperglycemic conditions, disrupt TET enzyme function, altering DNA methylation dynamics.

Metabolites derived from metabolic pathways intricately modulate DNA methylation processes. Acetyl-CoA, a central metabolite in cellular energy metabolism, emerges as a critical regulator of TET enzyme activity. Generated from pyruvate within the Krebs cycle, acetyl-CoA influences the function of TET enzymes, thereby shaping DNA methylation patterns (141).

Folate (Vitamin B9), an indispensable member of the B-vitamin complex, assumes a central role in DNA methylation by serving as a primary methyl donor. Its involvement in one-carbon metabolism, pivotal for methyl group supply, underscores its significance in epigenetic regulation. The folate cycle, primarily occurring in mitochondria, facilitates the synthesis of DNA and RNA building blocks while intricately linked to the methionine cycle, critical for S-adenosylmethionine (SAM) production. Genetic polymorphisms, such as the MTHFR C677T variant, disrupt folate metabolism, impacting DNA methylation patterns. Studies reveal dynamic aberrations in DNA methylation levels globally in response to folate intake, emphasizing the direct influence of dietary factors on epigenetic modifications. Research indicates that folate intake impacts DNA methylation levels in postmenopausal female lymphocytes. A folate-deficient diet led to decreased methylation, while a folate-rich diet increased it (147). In older women (60-85 years), a moderately folate-depleted diet resulted in reduced leukocyte DNA methylation, which didn’t significantly improve even with a folate-replete diet (148). A study also found that a 10-week folic acid supplement intervention increased DNA methylation in leukocytes by 31% (7). These findings imply that short-term folate depletion can reduce methylation of DNA globally, and recovery might be possible with folic acid supplementation, although its effectiveness may decrease with age.

Vitamin B12, another essential member of the B-vitamin complex, interfaces with DNA methylation through its involvement in one-carbon metabolism. Acting as a coenzyme for methionine synthase, vitamin B12 facilitates the conversion of homocysteine to methionine, a pivotal step in DNA methylation reactions. Kok (149) found that Numerous genes’ DNA methylation changed as a result of long-term folic acid and vitamin B12 supplements. After the intervention, there was a difference in the methylation of 163 sites from the baseline. Between the intervention and placebo groups, there were notable differences in six locations. Notably, alterations were discovered in the genes NODAL, ARMC8, and DIRAS3, which are connected to early embryonic development and carcinogenesis.

Moreover, vitamin B6 and riboflavin contribute to DNA methylation by supporting the folate cycle. These B vitamins aid in methyl group transfer, modulating DNA methylation dynamics and the profiles of gene expression. Choline and betaine, integral components of one-carbon metabolism, indirectly influence DNA methylation dynamics (150). Choline oxidation to betaine facilitates the maintenance of methionine homeostasis, crucial for DNA methylation reactions. Inadequate choline intake during pregnancy disrupts offspring DNA methylation patterns, highlighting the importance of maternal nutrition in epigenetic programming (151). Betaine also regulates homocysteine levels, crucial for preventing its accumulation and associated health complications. The intricate interplay between these metabolites underscores their collective impact on DNA methylation dynamics and ovarian aging processes (152).

Lastly, methionine, an indispensable amino acid, serves as a critical precursor of SAM, which is the main methyl donor in processes involving DNA methylation. Fluctuations in methionine levels exert profound effects on DNA methylation patterns, underscoring its pivotal function in the control of epigenetics. The production of SAM from methionine is essential for DNA methylation, as SAM is required by DNA methyltransferases (DNMTs) to catalyze the addition of methyl groups to DNA (150). Therefore, the availability of methionine influences the production of SAM and subsequently impacts DNA methylation levels. Increasing dietary intake of methionine has been speculated to potentially enhance DNA methylation by providing more substrate for SAM production.

The serine cycle, which is involved in *de novo* ATP synthesis, plays a crucial role in DNA methylation. Regardless of the methionine status, the serine cycle contributes to DNA methylation by providing the necessary ATP for the generation of SAM from methionine. This highlights the interconnectedness of various metabolic pathways in regulating DNA methylation processes.

### 5.2 Mitochondrial metabolite regulating histone modification

Histone modification is a key epigenetic mechanism that regulates gene expression. It involves the addition or removal of chemical groups to histone proteins, which are responsible for packaging DNA into a compact, organized structure called chromatin (9). The most common types of histone modifications include methylation, acetylation, phosphorylation, and ubiquitination (107). These modifications can alter the structure of the chromatin, thereby influencing gene accessibility and transcriptional activity. In the context of aging, changes in histone modifications have been observed in various organisms and tissues. For instance, alterations in histone acetylation and methylation patterns have been associated with ovarian aging (153). These findings suggest those histone modifications, and the enzymes that regulate them, could be potential targets for interventions aimed at modulating the aging process.

Amongst the metabolites that regulate histone acetylation, acetyl-CoA serves as the primary acetyl donor. Histone acetylation is catalyzed by histone acetyltransferases (HATs), which transfer an acetyl group from acetyl-CoA to specific lysine residues on histone proteins. The availability of acetyl-CoA is sensitive to nutrient availability and metabolic reprogramming, which can impact histone acetylation patterns and gene expression (154, 155). Acetyl-CoA is at the intersection of diverse metabolic pathways, and its availability is influenced by various metabolic processes. The crosstalk between metabolism and histone acetylation mediated by acetyl-CoA highlights the intricate relationship between cellular metabolism and epigenetic regulation. In 2021, Bradshaw examined that nuclear acetyl-CoA also plays a role in promoting longevity by increasing histone acetylation (156). Inhibitors of class I histone deacetylases (HDACs) increase longevity through increased histone acetylation.

Likewise, Acyl-CoA is a group of metabolic intermediates that play a crucial role in cellular metabolism and energy production. Acyl-CoA molecules serve as substrates for various enzymatic reactions, including fatty acid oxidation, fatty acid synthesis, and the tricarboxylic acid (TCA) cycle. Concentrations of different acyl-CoA metabolites in cells are tightly correlated with the relative abundances of acyl marks on histones. These metabolites act as signaling molecules and directly influence acylation dynamics in chromatin, linking cellular metabolism with epigenetic regulation (157). The Acyl-CoA synthetase long-chain family member 6 (ACSL6) gene has been connected to early ovarian failure (POF), according to a study (8). The study was conducted using genome-wide association research using dense single nucleotide polymorphisms as genetic markers and linkage disequilibrium-based methodology. The ACSL6 gene, located on chromosome 5q31, was found to be associated with POF, and disease-sensitive haplotypes were identified.

NAD+ is a critical coenzyme engaged in several cellular functions, including metabolism, DNA repair, and gene expression regulation. In the context of histone modifications, NAD+ has a major part in controlling the activity of sirtuins, a class of histone deacetylases that remove acetyl groups from histone proteins (158). NAD+ availability influences the deacetylation of histones by sirtuins, thereby affecting chromatin structure and gene expression. Changes in NAD+ levels can impact the ratio of acetylation to deacetylation in histones, leading to alterations in gene transcription and cellular responses to environmental cues. A recent study establishes causality between several age-related illnesses and a reduction in NAD+ levels. It also highlights promising results from strategies using NAD+ precursors to promote NAD+ biosynthesis, which could substantially improve oocyte quality and alleviate ovarian aging (159). In summary, NAD+ influences oocyte quality by enhancing energy production, DNA repair, and cellular resilience. Its anti-inflammatory effects and impact on SIRT1 activation contribute to alleviating ovarian aging. Researchers continue to explore NAD+ as a potential therapeutic avenue for reproductive health (160).

### 5.3 Mitochondrial metabolite regulating chromatin remodeling

The term “chromatin remodeling” describes the dynamic alterations in the composition and arrangement of chromatin, the complex of proteins and DNA that make up chromosomes in the nucleus of a cell. It involves the alteration of the accessibility of DNA to the cellular machinery, allowing for the regulation of the expression of genes and other genome-associated processes. This remodeling might not always be enzymatic and may use metabolites as regulators.

A by-product of amino acid metabolism, lipid metabolism, and glycolysis, methylglyoxal (MGO) can form covalent adducts with nucleophilic functional groups on histones and DNA. Chronic illnesses and aging are intimately associated with the production of MGO adducts known as advanced glycation end products (AGEs). MGO glycation of histones can destabilize the nucleosome structure and disrupt the landscape of chromatin modifications (161). A study focused on the accumulation of AGEs in the follicular fluid (FF), a liquid that surrounds the ovum in the ovaries. This fluid plays an important role in the maturation of the egg and its readiness for fertilization. The concentration of AGEs in the FF could potentially affect various aspects of assisted reproduction, such as ovarian responsiveness, embryo quality, follicular development, fertilization, embryonic development, and achievement of pregnancy (162).

In addition, 2-oxoglutarate has been found to encourage naïve embryonic stem cells (ESC cells to renew them) by increasing histone H3K27me3 demethylation. It has been observed that the addition of 2-oxoglutarate potentially increases the expression of pluripotency genes in induced pluripotent stem cell (iPSC) reprogramming by removing H3K9me3 and H3K36me3 marks (163). A study suggested that α-KG treatment can ameliorate detrimental effects on female rats’ fertility, potentially as a result of ovarian granulosa cell apoptosis being reduced and glycolysis being restored (164).

### 5.4 Mitochondrial metabolite regulating RNA modification

RNA modification refers to the process of chemically modifying RNA molecules, which can impact their structure, stability, and function. Methylation, which involves adding a methyl group to the RNA molecule, is one of the most prevalent RNA modifications. This modification is facilitated by a class of enzymes referred to as writers, which include proteins such as Mettl3, Mettl14, and Mettl16 (165).

m6A is a common RNA modification known as N6-methyladenosine. It is done when a methyl group is added to the adenosine base of RNA molecules. m6A modification is essential for controlling a variety of aspects of RNA metabolism, including stability of the RNA, splicing, translation, and degradation. It has a role in controlling the expression of genes. and cellular processes (165). m6A modification is reversible, and metabolites operate as substrates and/or allosteric regulators to directly affect the reaction’s flow. Metabolic processes, such as one-carbon metabolism and the tricarboxylic acid cycle, produce metabolites that affect the activities of m6A writers and erasers. For example, S-adenosyl methionine (SAM) is a methyl donor for m6A modification, while S-adenosyl homocysteine (SAH) inhibits writer activity (166).

A recent study suggests that m6A modification poses a significant role in female reproductive health and disease, and further research in this area could lead to improved understanding and potential therapeutic strategies (167).

## 6 Ovarian aging confers high risk of diseases

Hormonal, tissue, and cellular changes are all part of ovarian aging, which also includes a notable reduction in the number and quality of oocytes (174). These alterations have systemic impacts on many organs throughout the body in addition to impairing reproductive function. Decrease in important hormones like progesterone and estrogen impact many physiological functions and increase age-related diseases like osteoporosis, cardiovascular disease, and cognitive decline (175). Numerous age-related conditions, such as metabolic syndrome, osteoporosis, cardiovascular disease, cognitive decline, and several malignancies, are brought on faster by menopause (176). These diseases are more likely to occur during the transition to menopause, which is characterized by a considerable decrease in estrogen levels. After menopause, the risk of cardiovascular disease increases two to three times, annual bone loss is one to two percent, and 30 to 60 percent of postmenopausal women have metabolic syndrome (177). Postmenopausal women also have a significantly increased risk of mood disorders, ovarian and breast malignancies, and cognitive deterioration. These figures highlight how critical it is to manage the health issues that come with aging and menopause in women (178). Thus, beyond fertility, ovarian aging has a wider impact on women’s aging process by gradually lowering their quality of life and health (175).

POF is a significant health concern that brings about the cessation of ovarian function before the age of natural menopause. It affects up to one in 100 women, including one in 1000 under thirty years old (179). This condition is associated with a multitude of health problems and complications. One of the most pressing issues associated with POF is infertility, as the premature dissipation of ovarian activity leads to a decrease in the number of viable eggs, making natural conception a challenge. In addition to fertility issues, women with POF experience a hormonal imbalance characterized by lower levels of estrogen (180). This imbalance can manifest in several signs and symptoms, including vaginal dryness, nocturnal sweats, hot flashes, and decreased sexual desire. Moreover, this hormonal imbalance can lead to mood changes, including depression and anxiety (181).

Furthermore, low estrogen levels associated with POF can increase the risk of several health aberrations, such as osteoporosis, urogenital atrophy, cardiovascular disease, and hypothyroidism (180, 182). The diagnosis of POF can also have a significant psychological impact, leading to feelings of anger, depression, and anxiety, and can affect a woman’s self-esteem and body image.

Lastly, the symptoms associated with POF and the resulting infertility can significantly impact a woman’s quality of life. In conclusion, POF is a complex condition with far-reaching implications for a woman’s health, fertility, and overall quality of life. To comprehend this illness better and create efficient remedies, further research is required (183).

Gilmer (184), described how a drop in estrogen levels increases the risk of cardiovascular diseases as the said hormone is necessary to maintain healthy blood vessels and cholesterol levels. Furthermore, estrogen also possesses neuroprotective effects, and its decline during menopause is linked to a higher risk of neurodegenerative illnesses and cognitive impairment as seen in Alzheimer’s disease. It is also associated with an increase in the risk of musculoskeletal disorders such as osteoporosis and osteoarthritis. An essential component to retaining bone density is estrogens., and its decline during menopause can contribute to bone loss and increased fracture risk. Menopausal women are also at an increased chance of developing metabolic diseases like diabetes and obesity as hormonal changes can affect insulin sensitivity and metabolism, leading to disruptions in blood sugar regulation.

Further, delaying the process of ovarian aging can hold potential benefits (140). It can extend the reproductive lifespan of women, providing more time for family planning (123, 185). Women with delayed ovarian aging may experience a slower decline in reproductive function and may have a higher chance of maintaining regular menstrual cycles, endocrine homeostasis (186), and hormonal balance (187). They are also at a lower risk for a range of conditions, including cardiovascular disease, dementia, retinal disease, depression, and osteoporosis. It may also be associated with a lower risk of certain age-related diseases, such as breast and ovarian cancer. Studies have shown that genetic factors linked to delayed ovarian aging, such as BRCA mutations, may confer benefits in terms of enhanced fertility and improved fitness (3). Additionally, delayed ovarian aging may have evolutionary advantages. It may allow for the selection of higher-quality oocytes and embryos, leading to improved reproductive success and offspring quality (185).

Several pharmacological strategies have been explored to help delay ovarian aging which include antioxidants such as vitamins C, E, N-acetyl-L-cysteine, and proanthocyanidin; coenzymes Q10 to help with mitochondrial dysfunction and calorie restriction mimetics like metformin to prolong the delaying process (188). These work by following different mechanisms such as reducing oxidative stress using anti-oxidants, targeting mitochondrial dysfunction, mimicking calorie restriction strategies, using telomerase activators, and inhibiting the mTORC1 pathway to prevent over-activation of the primordial follicle pool, which is associated with ovarian aging (189).

## 7 Strategies to preserve ovarian function for ovarian longevity

**Figure 5:**
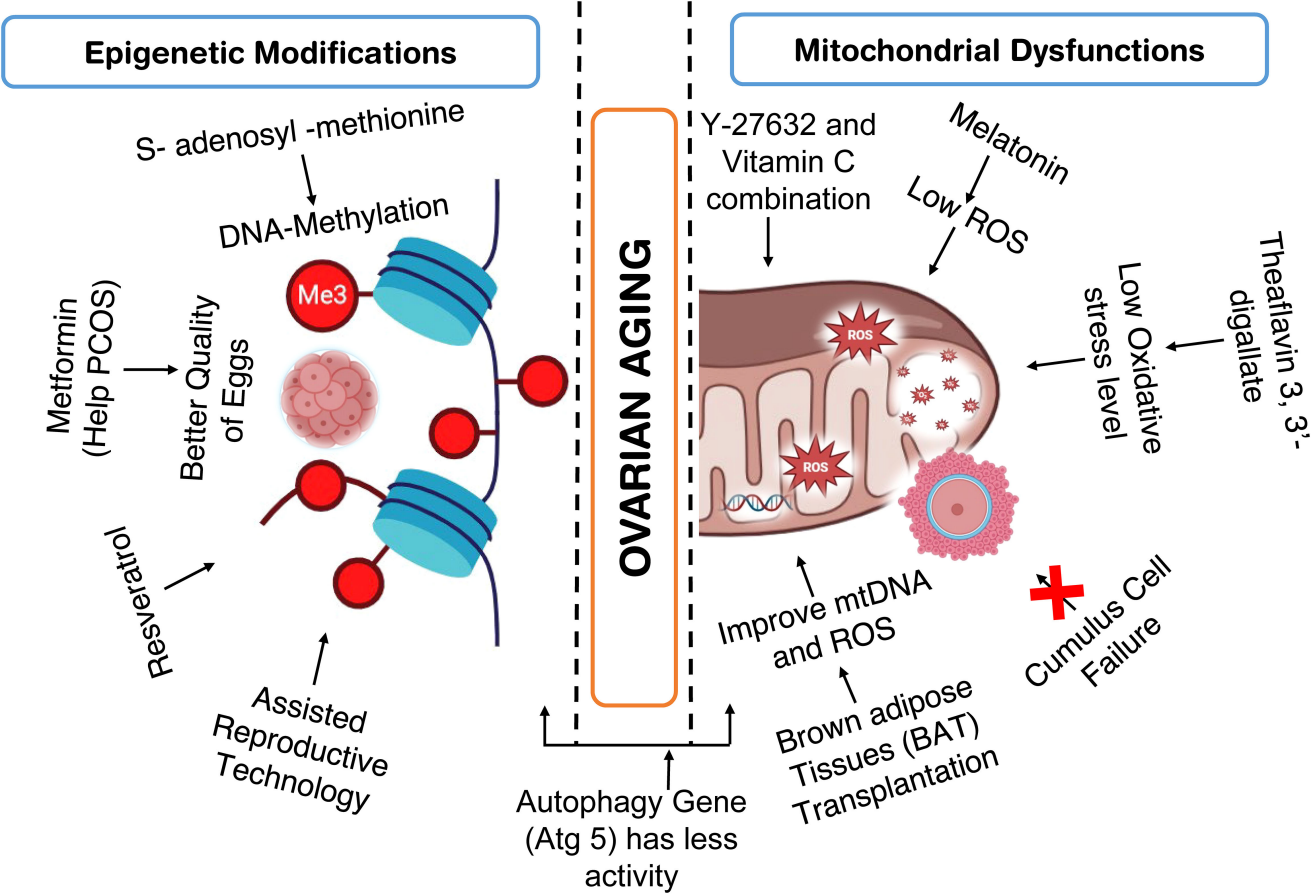
Studies conducted to improve ovarian health by addressing epigenetic modification and mitochondrial dysfunction.

As per the previous discussion, multiple factors such as Genetic alterations, epigenetic factors, ROS and oxidative stress, aneuploidy, mitochondrial dysfunction, and telomere shortening are responsible for ovarian aging. However, mitochondrial dysfunction and epigenetic alterations seem to be some of the potential factors responsible for ovarian aging. Hence in the recent past, a significant number of studies have been conducted to alter the cellular energy and/or epigenetic profile of ovarian cells (**Figure 5** **).**

### 7.1 Targeting mitochondria energetics of ovarian cells

Mitochondria are critical targets for preserving ovarian function (6, 190). Potential the mitochondrial membrane potential and mitochondrial oxidative activity complex decline with age, as do endogenous antioxidant system activities, indicating that redox imbalance plays a more prominent character in mature cells and tissues (191).

Melatonin treatment reduces the production of mitochondrial reactive oxygen species (mROS), prevents cell death, stops the collapse of the mitochondrial membrane potential, and maintains the functions of the respiratory chain complex in ovarian mitochondria (192). As melatonin, a circadian hormone reduces oxidative stress and inflammatory reactions to enhance the ability of endometrium to respond and preserve uterine homeostasis (2). Because of its anti-inflammatory and antioxidant qualities, melatonin is recognized to offer anti-aging benefits (193). In addition to controlling the body’s seasonal and circadian rhythms, melatonin (MT) can also delay the aging of ovaries, control their biological rhythm, encourage the development of follicles, and enhance the quality and rate of fertilization of oocytes. Additionally, the effects of MT on a number of ovarian-related disorders were examined, with ovarian aging and polycystic ovary syndrome (PCOS) receiving special attention (194). Supplemental melatonin use may help prevent infertility later in life, ease the frustration of women who are delaying having children, lessen the need for IVF-ET procedures, and assist in resolving the issue of non-aging-related infertility in women for the duration of their reproductive years (193). Female mice given melatonin showed increased mitochondrial antioxidant activity, lowering the risk of oxidative damage of the mitochondria caused by free radicals (192), Notably, melatonin administration increases SIRT3 activity and enhances the binding capability of FoxO3a to the regulators of both enzymes, namely superoxide dismutase 2 (SOD2) and catalase (CAT) (192).

Comparably, Theaflavin 3, 3’-gallate (TF3), extracted from black tea and serves as a bioactive polyphenol, possesses antioxidant qualities that may aid in maintaining the primordial follicle pool, restoring the estrous cycle, and boosting the number of progenies in old mice (190). TF3 exhibits a positive correlation with oocyte retrieval, oxidative stress levels, and spindle abnormalities. These findings suggest that TF3 might positively affect female reproduction and ovarian function by delaying ovarian aging, as evidenced by its ability to maintain ovarian reserve, shorten the estrous cycle, and increase litter size (190).

A study examined the relationship between aging-related declines in ovarian activity and fertility and the mitochondrial function of cumulus cells (CCs). Researchers discovered that older age groups of women with reduced ovarian reserve contained less and lower-quality eggs recovered. Their cumulus cells also had reduced energy output, altered structural characteristics, and decreased activity all indicators of mitochondrial malfunction. This study suggests that a major contributing element to the age-related loss in female fertility may be a mitochondrial failure in cumulus cells (195, 196).

Recent studies indicate that Vit C and Y27632 directly affect mitochondrial biogenesis and enhance their ability to maintain the youth of cellular aging. These agents were employed to dual-control both the metabolism of energy and mitochondrial mass. Y-27632 and vitamin C combination were found to effectively improve the quality of old cells (197). Increasing mitochondrial fusion appears to mitigate excessive mitochondrial fragmentation in old cells, reduces oxidative stress, and maintains mitochondrial membrane potential (197).

### 7.2 Approaches to alter the epigenetic profile of ovarian cells

Bacon reported that age and reproductive senescence are associated with a decrease in global DNA methylation. While supplementing with the S-adenosyl-methionine precursor methionine delayed the onset of perimenopause, treatment with the DNA-methyltransferase-1 inhibitor expedited the shift to reproductive senescence or menopause. DNA methylation of genes necessary for melatonin and circadian pathways, hormone and glutamate signaling, and other signaling pathways was found through genome-wide epigenetic study. Certain epigenetic modifications in these signaling pathways shed light on the cause of neurological disorders linked to perimenopause, like insomnia (198).

Further studies looked into the possibility of a connection between aging rats’ declining ovarian function and the cellular housekeeping mechanism of autophagy (199). They measured hormone levels, the activity of autophagy genes, and DNA methylation, a sign of gene silencing, to compare young, middle-aged, and elderly rats. The study discovered that all of the autophagy genes they looked at had less activity and that older rats had a lower level of estrogen. Notably, the age-related rise in methylation of the DNA was observed in the autophagy gene Atg5. This implies that autophagy in aging ovaries may be suppressed by DNA methylation, which could lead to a lack of ovaries’ ability to function (199).

Olsen determined the epigenetic profiles of mural granulosa cells (MGCs), which are involved in follicle development, from women with varying levels of ovarian reserve (normal, diminished, and high). They compared DNA methylation patterns between groups and found that MGCs from women with diminished ovarian reserve showed significantly higher variability in DNA methylation compared to the control group; these variable sites were enriched in genes essential for the development of follicles. However, leukocytes, or white blood cells, did not show any differences between groups concerning ovarian reserve. This suggests that decreased ovarian reserve is linked to particular changes in the epigenetic profile of MGCs, which may be linked to premature aging in these cells (200).

Another study analyzed the potential benefits of metformin on egg quality in mice using a DHEA-induced PCOS model. Treatment with metformin seemed to offset the effects, of DHEA possibly through lowering epigenetic changes and oxidative stress. This shows that metformin may be a useful medication to help PCOS-affected women produce better-quality eggs (201).

Resveratrol can mitigate altered DNA methylation and decreased gene activity in the ovaries of aging mice. However, the research also showed that Tet2, a gene, is necessary for these improvements. Tet2-deficient mice did not benefit from resveratrol treatment in the same ways. This implies that Tet2 is necessary for the action of resveratrol (202).

An additional study by (191) examining the relationship between epigenetic senescence and infertility found that mothers who conceived using Assisted Reproductive Technology (ART) aged more quickly than mothers who conceived naturally.

## 8 Conclusions

The ovary is the first organ to age in the women’s body, affecting fertility and driving overall health and disease in women. In addition to natural ovarian aging in all women, premature ovarian insufficiency (POI) is reported in up to 2% of women, amounting to about 80 million worldwide. Indeed, germ-line mutations in at least eight nuclear genes, including POLG1, known to perform mitochondrial function, are reported in women suffering from POI (203) (204).

The defective mitochondria are the most significant marker for aging (1). Additionally, the mtDNA content in oocytes is also reported to decline with age (6). The content of mtDNA in the oocytes is an important criterion behind the fertilization of oocytes as well as embryonic development. mtDNA is mostly lower in the oocytes of aged women or those with diminished ovarian reserve, in comparison to those of less age and have normal ovarian reserve (205). Diminished ovarian reserve is also associated with decreased mtDNA content in the cumulus cells compared to that in women with normal ovarian reserve (71). The mtDNA content is also reported to be lesser in the unfertilized oocytes of women having different infertility problems (206). Similarly, oocytes of cows older than 15 years are also reported to have a significantly lower copy number of mtDNA than oocytes of younger animals (207). The low copy number of mtDNA is also observed in poor responders to IVF. In physiologically menopausal women, mtDNA levels are lower than normal (77). Based on the positive association between the mt DNA content of oocytes and developmental proficiency and the evidence discussed in this article, we envision that the increase in mitochondrial biogenesis through various pharmacologic interventions may have a significant effect on ovarian function and longevity. A comprehensive and strategic effort is required to screen and identify agents that protect, preserve, or promote mitochondrial function in oocytes to preserve ovarian function, delay menopause, and provide better health outcomes.
